# Validity and prognostic significance of sperm protein 17 as a tumor biomarker for epithelial ovarian cancer: a retrospective study

**DOI:** 10.1186/s12885-018-4880-x

**Published:** 2018-10-11

**Authors:** Laurie L. Brunette, Paulette Y. Mhawech-Fauceglia, Lingyun Ji, Joseph G. Skeate, Heike E. Brand, Kate Lawrenson, Saloni Walia, Maurizio Chiriva-Internati, Susan Groshen, Lynda D. Roman, W. Martin Kast, Diane M. Da Silva

**Affiliations:** 1 Department of Obstetrics & Gynecology, University of Southern California, 1450 Biggy Street, Los Angeles, CA 90033 USA; 2 Department of Pathology, University of Southern California, California, Los Angeles USA; 3 Norris Comprehensive Cancer Center, University of Southern California, California, Los Angeles USA; 4 Department of Molecular Microbiology & Immunology, University of Southern California, California, Los Angeles USA; 5 Department of Obstetrics & Gynecology, Cedars-Sinai Medical Center, California, Los Angeles USA; 6 Department of Myeloma and Lymphoma, MD Anderson Cancer Center, University of Texas, Houston, TX USA; 7 Kiromic, Inc, Houston, TX USA; 8 Department of Preventive Medicine, University of Southern California, California, Los Angeles USA

**Keywords:** Epithelial ovarian cancer, Borderline ovarian tumor, Sperm protein 17, Ovarian biomarker, Serous

## Abstract

**Background:**

Prior small studies have shown increased expression of sperm protein 17 (Sp17) in epithelial ovarian cancer (EOC) tissue and suggest Sp17 as a potential biomarker for EOC. However, how Sp17 expression varies with histology, grade, and stage of EOC and its expression in other ovarian neoplasms has not been defined. It is unknown whether patients with EOC have elevated serum Sp17 levels or if Sp17 expression is associated with survival outcomes.

**Methods:**

The study included 982 patients with benign, borderline, and malignant ovarian neoplasms and normal ovary. There were 878 patients with tissue only, 39 with serum only, and 65 with matching serum and tissue. Immunohistochemical (IHC) staining with anti-Sp17 antibody was performed on tissue specimens and the intensity scored as weak, moderate, or strong. A sandwich enzyme-linked immunosorbent assay (ELISA) was performed to measure Sp17 sera concentrations.

**Results:**

Sp17 expression was most commonly seen in serous cystadenomas (83%) and serous borderline tumors (100%). Of the 773 EOC specimens, 223 (30%) expressed Sp17. Grade and histology were significantly associated with Sp17 expression among EOC specimens (*p* < 0.001) on both univariate and multivariable analysis, with grade 1 serous adenocarcinomas showing the highest expression (51%). Sp17 expression was limited in other benign and non-epithelial malignant neoplasms. Neither Sp17 tissue expression nor serum concentration correlated with survival outcomes. Serum concentrations were higher in patients with Sp17 tissue expression, and the highest concentrations were noted among patients with serous and clear cell adenocarcinomas.

**Conclusions:**

Sp17 is highly expressed in benign, borderline, and low grade malignant serous ovarian neoplasms and can be quantified in serum. Sp17 expression may have diagnostic significance in this subset of patients.

**Electronic supplementary material:**

The online version of this article (10.1186/s12885-018-4880-x) contains supplementary material, which is available to authorized users.

## Background

Epithelial ovarian cancer (EOC) is the fifth most common cause of female cancer-related deaths. When detected at an early stage, EOC has a 5-year overall survival of over 90%. Unfortunately, only 15% of all EOC is detected as localized disease, mostly due to the lack of clinical symptoms and the absence of reliable early detection biomarkers. As a result, a majority of EOC is diagnosed at an advanced stage when 5-year overall survival is below 30% [1]. At present, serum CA-125 is the most extensively used biomarker to preoperatively discriminate between benign and malignant ovarian neoplasms, detect recurrence, and monitor response to treatment. However, approximately 1% of healthy women and 6% of women with benign lesions have an elevation in CA-125 [2]. Human epididymis protein 4 (HE4) appears to have similar diagnostic performance as CA-125 [3], but is currently only approved for monitoring women with EOC for disease recurrence or progression. New biomarkers that have roles in screening, therapy, or improved diagnostic or prognostic performance are urgently needed.

One class of proteins with high potential as oncologic biomarkers are cancer-testis antigens (CTAs). CTAs are defined by a) high levels of expression in male germ cells, b) lack of expression in normal somatic tissues, and c) aberrant expression in a variety of tumor types [4, 5]. One CTA that has been proposed as a potential biomarker for ovarian cancer is sperm protein 17 (Sp17). Sp17 is only expressed in humans in the flagella of spermatozoa and in the ciliated cells of the respiratory tract, fallopian tube, and male efferent ducts [6, 7]. Non-ciliated cells do not express the protein, including other cell types within the respiratory and reproductive tracts [7]. The function of Sp17 is not yet fully understood, but the protein’s presence in cilia and flagella suggests a role in the regulation of motility. Sp17 was also found to bind extracellular heparin sulfate and may play a critical role in cell/cell adhesion and cell migration [8].

Prior small studies report anywhere from 20 to 79% of EOC specimens express Sp17, while normal ovarian epithelium does not [6, 9–13]. In order to further evaluate the overall validity of Sp17 as a biomarker for EOC, more complete data are needed with regards to Sp17 tissue expression across the different histologies, grades, and stages. It is also pertinent to define Sp17 expression in other benign and malignant adnexal masses which can mimic the radiographic appearance and symptomatology of EOC. Moreover, one of the aims of this study was to assess the feasibility of measuring Sp17 serum concentrations in EOC patients for the first time and compare these levels to tissue expression. Therefore, the objectives of this study were threefold: 1) to characterize Sp17 tissue expression in a large cohort of benign and malignant ovarian neoplasms of various histologies and grades, 2) to determine its association with recurrence and survival outcomes, and 3) to correlate Sp17 tissue expression to its serum levels.

## Methods

### Specimen acquisition

Tissue specimens were sourced from two institutions and supplemented with commercially available tissue microarrays (TMAs). Inclusion criteria for specimens included any primary EOC (primary peritoneal and fallopian tube carcinomas were included in this category), borderline ovarian tumors (BOTs), benign ovarian neoplasms, sex cord stromal tumors (SCSTs), and germ cell tumors (GCTs). Samples of normal ovary and fallopian tubes were also tested for Sp17 expression. Metastatic carcinoma to the ovary was excluded as were adnexal masses that were not ovarian neoplasms (i.e. hydrosalpinx, tubo-ovarian abscess, etc). Any patient who had previously received chemotherapy for an ovarian malignancy was excluded.

The project was approved by the University of Southern California Institutional Review Board (IRB) (#HS-13-00782; HS-13-00268). There were 982 patients in the study, including 187 from Los Angeles County + University of Southern California (LAC+USC) Medical Center, 286 from the Oregon Health System, and 509 that were purchased commercially.

As part of an ongoing tissue collection study (The USC Department of Obstetrics & Gynecology Gynecologic Tissue and Fluid Repository) at LAC+USC Medical Center, all patients undergoing surgery with the gynecologic oncology service from 2007 to 2014 had blood and tissue collected on the day of surgery following written informed consent. Of these 187 patients, 39 had only serum available for evaluation and 83 had only tissue available. The remaining 65 patients had both tissue and serum available for analysis. Of those cases with tissue available for immunohistochemistry (IHC), 109 were made into TMAs and 39 were examined as whole specimens including six normal ovaries or fallopian tubes.

The 286 specimens from the Oregon Health System were collected from 1992 to 2011 after institutional IRB approval and made into TMAs. The commercially available TMAs (Catalog #OV2085-9US, US Biomax, Inc., Rockville, MD, USA) included twelve normal ovarian or fallopian tube specimens and 497 benign or malignant ovarian neoplasms. All TMA specimens were represented in duplicate as 1 mm punches.

### Clinical data collection

Clinical data for the commercial TMA specimens was provided by US Biomax, Inc., and included patient age, histology, stage, and grade. All other tissue specimens collected from the two institutions were examined by an experienced pathologist at the time of surgery. The neoplasms were classified according to World Health Organization criteria [14] and analyzed for histology and grade if appropriate. Grades for all EOC specimens were reported using the Silverberg three-tiered grading system [15]. Patients were surgically staged according to International Federation of Gynecology and Obstetrics (FIGO) standards. Prior to January 1, 2014, the 1988 FIGO staging system was used. After this date, the updated 2014 FIGO staging system was used [16]. Stage was analyzed as a whole-number discrete variable (I, II, III, and IV), thus, the minor differences in the two staging criteria systems over time were not significant. Additional clinical information including data on recurrence, treatment, and survival were collected retrospectively from the patients’ medical records.

### Processing of serum samples

Serum samples were processed within four hours of collection by the institutional translational pathology lab in a standardized manner. Blood samples were collected after anesthesia but before surgery and held at room temperature for clotting. Serum was collected after centrifugation at 2000×g for 10 min, aliquoted and then stored in a -80 °C freezer until use.

### IHC staining of tissue for Sp17 expression

IHC for Sp17 tissue expression was performed on the pre-made TMAs or on 4 μm whole mount tissue sections from formalin-fixed paraffin embedded tissue. The TMA or whole mount slides were incubated with affinity purified rabbit polyclonal antibody to Sp17 (Proteintech, Rosemont, IL, USA) used at a 1:300 dilution following standard deparaffinization in xylene and heat-induced antigen retrieval in citrate buffer, pH 6.0. Sp17 antibody binding was detected using the ImmPRESS HRP Universal Antibody (anti-mouse IgG/anti-rabbit IgG) Peroxidase Polymer Detection Kit (Vector Labs, Burlingame, CA, USA) followed by 3–3′-Diaminobenzide (DAB) chromogen substrate addition. Polyclonal non-immune rabbit IgG was used as a negative control on testis tissue and on select whole tissue ovarian cancer specimens to confirm specificity of Sp17 staining. Slides were counterstained with Gill’s hematoxylin, dehydrated, and mounted. Human testicular tissue served as a positive control for Sp17 staining. Tissue from various non-reproductive organs served as a negative control.

### Scoring of tissue specimens for Sp17 expression

Evaluation of IHC staining was determined by a single expert gynecologic oncology pathologist (PMF) who was blinded to the clinical data. Any Sp17 expression in the epithelial or neoplastic cells of interest was considered a positive result. The intensity of staining was scored as weak, moderate, or strong. A subset of duplicate cores was analyzed and showed 97% concordance for Sp17 staining. If one of the TMA specimens was negative and the other positive, the results from the positive specimen were included, as the negative specimen was thought to be negative due to sampling error. If there was a discrepancy in scoring between two positive specimens, the score for the weaker staining specimen was used.

### Sp17 enzyme-linked immunosorbent assay (ELISA)

Human sperm surface protein Sp17 ELISA kits (Cusabio Biotech, Wuhan, China) were used to measure the Sp17 serum concentrations. The serum was tested per the manufacturer’s specifications. In brief, the Sp17 assay is a sandwich ELISA with anti-Sp17 antibody coated on the microplate. All serum samples were thawed to 4 °C, diluted 1:2 in sterile deionized water, and 50 μL/well was added in duplicate to the microplate. Standards of Sp17 protein of known concentration were also added to the plate in duplicate. 50 μL of Horseradish peroxidase (HRP)-conjugate was added to each well, and the plate was incubated for one hour at 37 °C. Wells were washed thrice with wash buffer, followed by addition of the two provided substrates, and the plate was incubated at 37 °C for fifteen minutes. The stop solution was added and the optical density of each well was measured with a Chameleon V multifunction plate reader (BioTek Instruments, Winooski, VT) at 450 nm. The Sp17 serum concentration of each well was calculated using the standard curve created by the standards on the same plate. The values of the two wells were averaged to give the final concentration.

### ONCOMINE data

Publicly available microarray data sets were queried from the ONCOMINE database (www.oncomine.org) using the following search terms: cancer type: ovarian cancer, and gene: SPA17. Primary sources which contained data for ovarian cancer grade and borderline ovarian surface epithelial stromal tissue were selected for further analysis. As a result, two datasets which included patients with ovarian serous adenocarcinoma were retrieved from the ONCOMINE database: Tothill et al. [17] and Anglesio et al. [18].

### Statistical analysis

Analyses were performed separately for three cohorts of patients: 1) patients who had Sp17 tissue expression scores from IHC, 2) patients who had serum Sp17 concentrations, and 3) patients who had both Sp17 tissue expression scores and serum Sp17 concentrations. For Cohort 1, analysis was performed to examine the association between Sp17 tissue expression and patient or disease characteristics, as well as the association between Sp17 tissue expression and patients’ failure free survival (FFS) or overall survival (OS). Associations between serum Sp17 levels and patient/disease characteristics or patients’ FFS and OS were assessed using Cohort 2. The correlation between Sp17 tissue expression and Sp17 serum concentration was tested using Cohort 3.

FFS was defined as the time between date of surgery and date of recurrence or death, if death occurred prior to recurrence; patients who had persistent disease after treatment were coded as having an event for FFS at one month after surgery; patients who were alive without recurrence, were censored at the last follow-up date. OS was defined as the time between date of surgery and date of death by any cause; patients who were alive, were censored at the last follow-up date. Fifteen patients were known to have died, but date of death was unknown. In our analyses, those patients were censored on the date they were last known to be alive. Patients who did not have follow-up data were excluded from the FFS or OS analyses.

The associations between Sp17 tissue expression and patient or disease characteristics were evaluated using logistic regression models; odds ratio’s (OR’s), used to quantify the size of the association, were calculated based on the regression parameter estimates in the logistic model. FFS and OS probabilities were calculated using Kaplan-Meier methods with Greenwood standard errors. The associations between Sp17 tissue expression and patients’ FFS or OS were examined using parametric survival models assuming a Weibull survival distribution; hazard ratio’s (HR’s), used to quantify the size of the association, were calculated based on the regression parameter estimates in the survival model. An intraclass correlation was estimated for tumor samples from the same TMA sources, and the effect of TMA sources was treated as a random effect in the logistic regression analyses and the survival analyses.

Analysis of Variance (ANOVA) was used to compare serum Sp17 concentrations among ovarian neoplasms of different disease categories or histology types, or between neoplasms with positive versus negative IHC results. For comparisons that were made among more than two groups, when the global test was statistically significant, Tukey’s honest significance difference (HSD) test with familywise Type I error of 0.05 was used for pairwise comparisons. Tukey–Kramer adjustment was done to account for the unbalanced sample sizes among the groups. Correlations between measured serum Sp17 concentrations and clinically available CA-125 and HE4 concentrations were calculated using Spearman’s correlation coefficient. Serum Sp17 levels were transformed on the log 10 scale before analyses were performed.

Log_2_ transformed Oncomine source data were used to compare the Sp17 messenger ribonucleic acid (mRNA) expression levels between borderline tumors and carcinoma (for the Tothill and Anglesio data) and to compare the expression levels by grade (Tothill data). A 2 × 2 ANOVA was used to compare the log_2_(Sp17) by dataset source and borderline tumors versus carcinoma; a one-way ANOVA was used to compare the log_2_(Sp17) by grade; pairwise comparisons were performed as described above.

All *p*-values reported are two sided. Statistical analyses were performed using STATA software (version 11.0; StataCorp LP College Station, TX). GraphPad PRISM (V6.0) was used to analyze ONCOMINE data.

## Results

There were 982 patients included in the study, of whom 878 had tissue specimens only, 39 had serum specimens only, and 65 had both. The median age was 53 years old (range: 11–89). Eighteen patients had normal ovarian or fallopian tube tissue available. Fifteen patients had a benign ovarian neoplasm, and 34 had a BOT. Sex cord stromal tumors (SCTs) and germ cell tumors (GCTs) were seen in 64 and 49 patients, respectively. The remaining 802 patients had EOC. Of the patients with EOC, 66% had serous histology, 9% endometrioid, 7% clear cell, and 10% mucinous. This closely resembles the distribution of histologies in the general population (70–80, 10, 10, 3% respectively) [14]. Our cohort includes a higher percentage of early stage (48%) and high grade (80%) compared to the general population (34 and 67% respectively) [1, 19]. Complete demographic data are shown in Table 1.

**Table 1:** Patient characteristics

	Whole cohort	Patients with tissue specimens	Patients with serum specimens
	Number of specimens, n (%)		
Age in years	982 (100)	943 (100)	104 (100)
0–19	24 (2)	24 (3)	1 (1)
20–39	133 (14)	125 (13)	16 (15)
40–59	510 (52)	487 (52)	64 (62)
60+	306 (31)	298 (32)	23 (22)
Unknown	9 (1)	9 (1)	0 (0)
Normal ovary	15	15	0
Normal fallopian tube	3	3	0
Benign ovarian neoplasms	15	11	7
Serous cystadenoma	6	6	1
Mucinous cystadenoma	2	2	1
Other	7	3	5
Borderline ovarian tumors	34	33	15
Serous	26	26	9
Mucinous	7	6	5
Other	1	1	1
Sex cord stromal tumors	64	61	5
Germ cell tumors	49	47	2
Epithelial ovarian carcinoma	802 (100)	773 (100)	75 (100)
Grade			
1	133 (17)	132 (17)	23 (31)
2	212 (26)	211 (27)	4 (5)
3	437 (54)	420 (54)	48 (64)
Unknown	20 (2)	10 (1)	0 (0)
Stage			
I	289 (36)	283 (37)	26 (35)
II	93 (12)	91 (12)	6 (8)
III	279 (35)	262 (34)	35 (47)
IV	53 (7)	50 (6)	8 (11)
Unknown or unstaged	88 (11)	87 (11)	0 (0)
Histology			
Serous	531 (66)	518 (67)	29 (39)
Endometrioid	73 (9)	68 (8)	14 (19)
Clear cell	58 (7)	56 (7)	8 (11)
Mucinous	79 (10)	72 (9)	12 (16)
Mixed/Other	61 (8)	59 (8)	12 (16)

### Sp17 is strongly expressed in ovarian neoplasms of serous histology

**Fig. 1:**
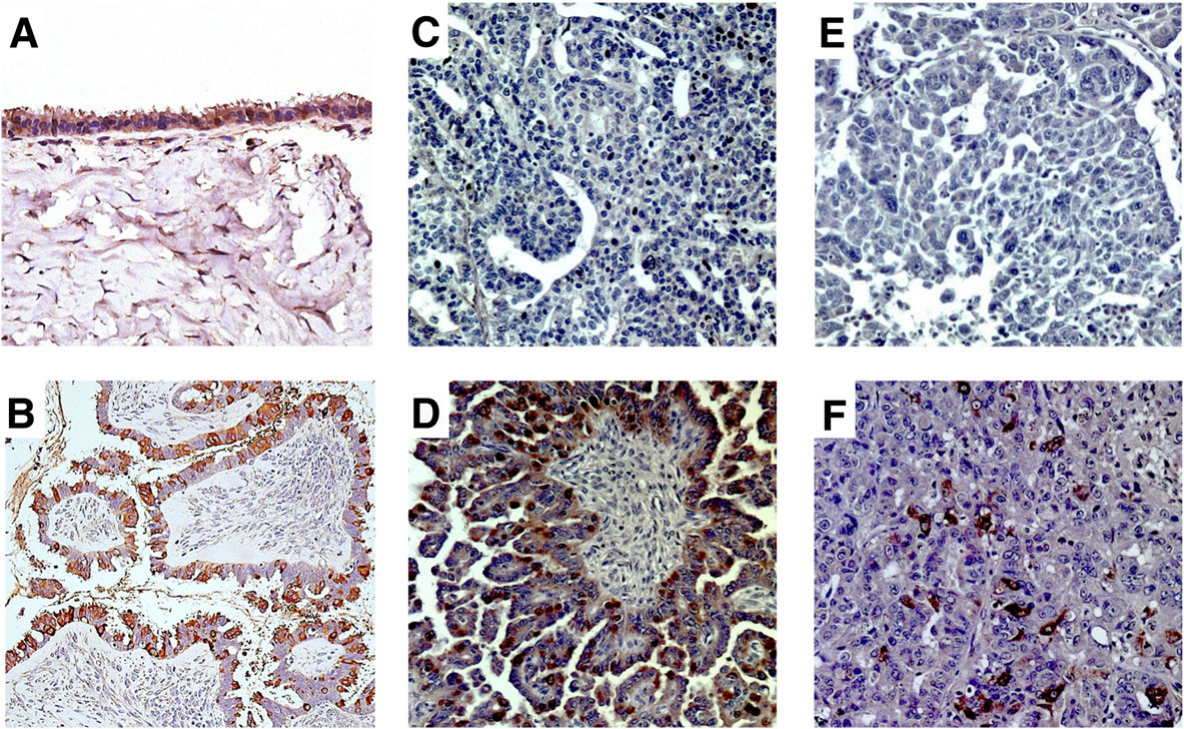
Representative Sp17 immunohistochemistry staining in ovarian tissue of serous histology. Examples of Sp17 protein expression in benign and malignant ovarian tissue demonstrating both positive and negative staining and intensity of Sp17 staining are shown. **a**) Serous cystadenoma showing tissue staining positive with strong expression of Sp17 in ciliated cells. **b**) Serous borderline tumor showing strong Sp17 expression. **c**) Grade 1 serous adenocarcinoma negative for Sp17 expression. **d**) Grade 1 serous adenocarcinoma showing strong Sp17 expression. **e**) Grade 3 serous adenocarcinoma negative for Sp17. **f**) Grade 3 serous adenocarcinoma demonstrating strong Sp17 staining. Stroma surrounding epithelial cells is negative

Sp17 protein expression was analyzed by IHC on 943 specimens that were assembled primarily on TMAs or as whole tissue mounts. The results are represented in Table 2. Sp17 was cytoplasmic and homogenous in its expression. All fallopian tube epithelium strongly expressed Sp17 (*n* = 3), but only 13% of the normal ovarian specimens were positive (*n* = 15). Sp17 expression was rare among GCTs (4%) and SCSTs (7%). Of the 11 benign ovarian neoplasms, 6 (55%) expressed Sp17. This included five of the six (83%) serous cystadenomas, which were all strongly positive. All 26 of the serous BOTs expressed Sp17 with 69% showing strong staining, but the other histologies of BOTs were negative for Sp17. Of the 773 EOC specimens, 223 (30%) were positive overall. Representative Sp17 staining of tissue sections is shown in Fig. 1. Normal testis tissue was used as a positive control for Sp17 staining and showed the expected Sp17 expression in the spermatocytes and mature sperm located within the seminiferous tubules and the lumen of the testis.

**Table 2:** Summary of tissue expression of Sp17 by type of epithelium or neoplasm

Disease Category	Sp17 staining (# positive/# total (%))
Normal fallopian tube epithelium	3/3 (100)
Normal ovarian epithelium	2/15 (13)
Benign ovarian neoplasms	6/11 (55)
Borderline ovarian tumors	26/33 (79)
Epithelial ovarian carcinomas	223/773 (30)
Sex cord stromal tumors	4/61 (7)
Germ cell tumors	2/47 (4)

Univariate and multivariable analyses of the EOC tissue specimens was performed to determine the association between Sp17 expression and age, histology, grade and stage of EOC (Table 3). On both univariate and multivariable analysis, patient age and stage were not correlated with Sp17 tissue expression. Grade was inversely correlated with Sp17 tissue expression, and this was highly statistically significant on multivariable analysis (OR = 0.41 for grade 2 and OR = 0.30 for grade 3, *p* < 0.001). Histology of the cancer was also significantly associated with Sp17 tissue expression, with serous histology showing the highest rates of expression (p < 0.001). Overall, 35% of serous carcinomas were found to be positive. Among these, 51% of grade 1 (*n* = 62), 35% of grade 2 (*n* = 135), and 31% of grade 3 (*n* = 315) showed Sp17 expression. This was statistically significant on both univariate and multivariable analyses (*p* < 0.001). Of the positive serous carcinoma specimens, strong Sp17 expression was seen in 46% of grade 1, 32% of grade 2, and 39% of grade 3. Endometrioid and clear cell adenocarcinomas showed the next highest expression of Sp17, while a smaller percentage of mucinous adenocarcinomas expressed Sp17. These results suggest that expression of Sp17 is differentially expressed by EOC tumors of low-grade serous histology as well as in benign and serous borderline ovarian tumors.

**Table 3:** Univariate and multivariable analysis of Sp17 tissue expression among epithelial ovarian carcinoma specimens

Variable	Number of patients^b^, n (% expressing Sp17^c^) (*N* = 773)	Univariate		Multivariable^a^	
		OR^d^ (95%CI)	*p*	OR^d^ (95%CI)	*p*
Age at Diagnosis (yrs)			0.53 ^2^		0.68 ^2^
0–39	75 (34)	1.0		1.0	
40–49	169 (29)	0.78 (0.40, 1.5)		0.91 (0.45, 1.8)	
50–59	251 (31)	0.85 (0.45, 1.6)		1.01 (0.52, 2.0)	
ε 60	273 (29)	0.76 (0.40, 1.4)		0.85 (0.43, 1.7)	
Unknown ^b^	5				
Grade			0.011 ^2^		< 0.001 ^2^
1	122 (40)	1.0		1.0	
2	211 (30)	0.57 (0.33, 0.97)		0.41 (0.23, 0.73)	
3	420 (28)	0.50 (0.30, 0.82)		0.30 (0.17, 0.53)	
Unknown ^b^	20				
Stage			0.24 ^2^		0.38 ^2^
I	282 (27)	1.0		1.0	
II	91 (29)	1.1 (0.63, 2.0)		0.94 (0.51, 1.7)	
III	262 (34)	1.4 (0.88, 2.4)		1.4 (0.78, 2.3)	
IV	50 (31)	1.2 (0.55, 2.6)		1.2 (0.53, 2.7)	
Unknown ^b^	88				
Histology			0.002 ^1^		< 0.001 ^1^
Serous	518 (35)	1.0		1.0	
Mucinous adenocarcinoma	72 (16)	0.28 (0.13, 0.59)		0.18 (0.08, 0.40)	
Endometrioid adenocarcinoma	68 (26)	0.58 (0.31, 1.1)		0.50 (0.25, 1.0)	
Clear cell	56 (24)	0.53 (0.27, 1.03)		0.63 (0.31, 1.3)	
Mixed/Other	59 (20)	0.38 (0.18, 0.80)		0.42 (0.19, 0.92)	

Abbreviations: *OR* – Odds Ratio; *CI* – Confidence Interval

^1^Overall *p* value

^2^*p* value from trend test

^a^The multivariable model included all the variables listed in the table

^b^ Patients with unknown values were included in the analysis using an unknown category

^c^% of patients with positive immunohistochemisty was estimated from logistic models that adjusted for TMA source, rather than using the simple weighted average method

^d^Odds ratio calculated based on the regression parameter estimates in the logistic model

### Sp17 RNA is significantly upregulated in borderline ovarian carcinomas compared to ovarian adenocarcinoma

**Fig. 2:**
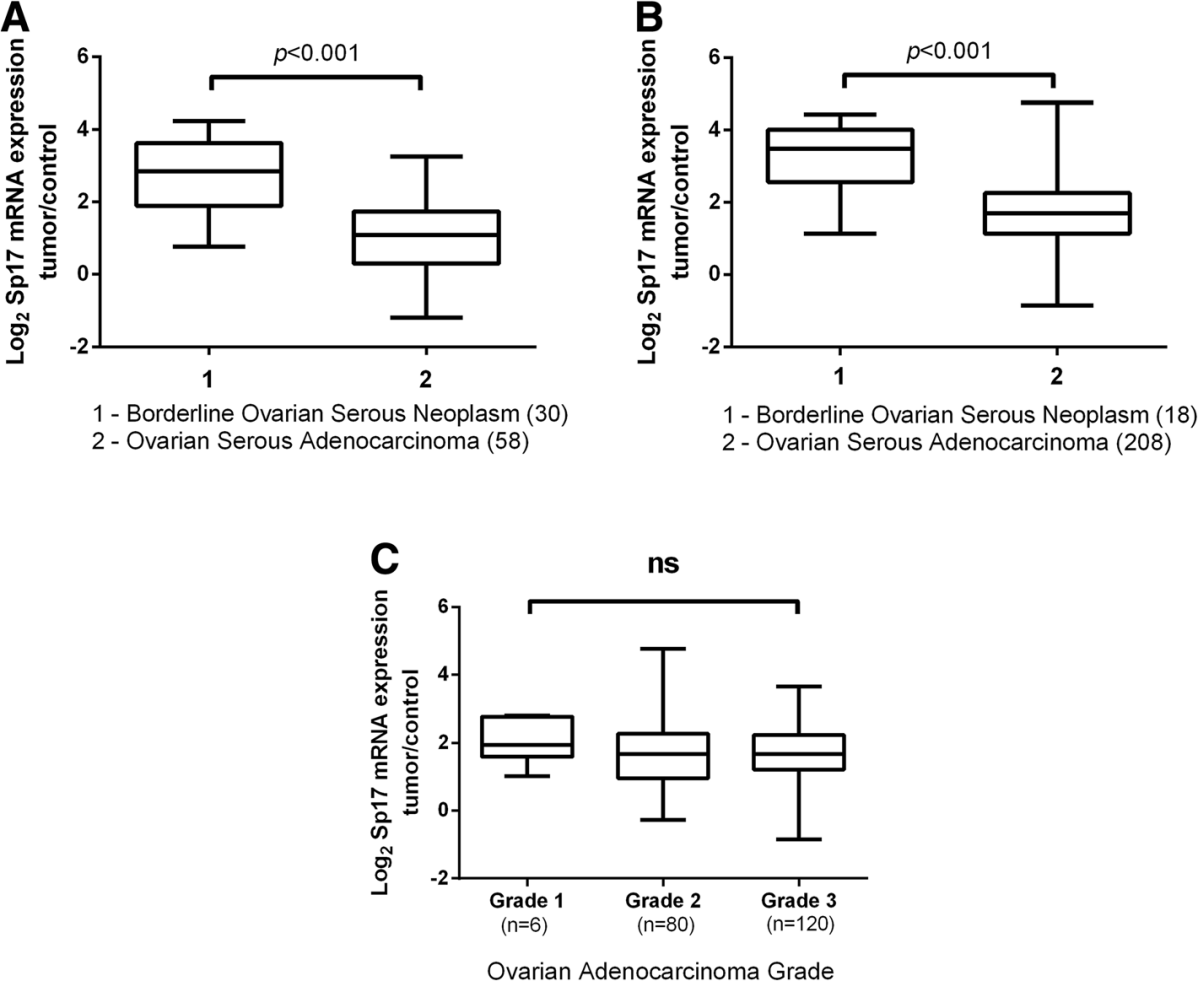
Sp17 RNA expression is upregulated in borderline ovarian cancers. Sp17 mRNA expression was analyzed from two publically available patient tumor Oncomine datasets. **a** Sp17 mRNA levels are significantly increased in borderline ovarian serous adenocarcinoma when compared to ovarian serous adenocarcinoma (*p* < 0.001) in Anglesio et al. dataset [18]. **b** Sp17 mRNA levels are significantly increased in borderline ovarian serous adenocarcinoma when compared to ovarian adenocarcinoma (p < 0.001) in Tothill et al. dataset [17] (**c**) No significant difference in Sp17 expression is seen between grades of ovarian adenocarcinoma (*p* = 0.42) [17]. Box encompasses 25th–75th percentile, solid black line indicates median and error bars indicate range. Numbers in parentheses indicate number of cases included in the analysis

Analyzing two sets of patient tumor microarray data from the ONCOMINE database [17, 18], we found that Sp17 mRNA expression levels followed the same trend as seen in our own investigation. Specifically, in both datasets serous BOTs had a significantly higher expression level of Sp17 mRNA when compared to serous adenocarcinomas (p < 0.001); the levels were not significantly different between the two datasets (*p* = 0.14 for dataset effect and *p* = 0.84 for interaction). When comparing Sp17 expression by grade of serous carcinoma in the Tothill et al. dataset [17] there was no statistically significant difference in expression levels (*p* = 0.42), possibly explained by the underrepresented number of grade 1 samples available (*n* = 6). (Fig. 2). These results support our protein expression findings and provide evidence that heightened Sp17 expression in BOTs can be seen in multiple, independently collected datasets.

### Prognostic significance of Sp17 expression in EOC

To determine whether Sp17 tissue expression was associated with clinical outcome, we analyzed FFS and OS among the 336 patients with EOC for whom we had clinical follow-up data. Median follow-up time among patients who were alive as of the last follow-up date was 24 months. Sp17 expression was not statistically significantly associated with FFS **(**See Additional file 1: Table S1) or OS (See Additional file 1: Table S2) on univariate or multivariable analyses. However, grade, histology, and stage were significantly associated with FFS on multivariable analysis as expected **(**Additional file 1: Table S1). Grade and stage remained significantly associated with OS on multivariable analysis **(**Additional file 1: Table S2).

### Sp17 is detectable in serum of ovarian cancer patients

**Fig. 3:**
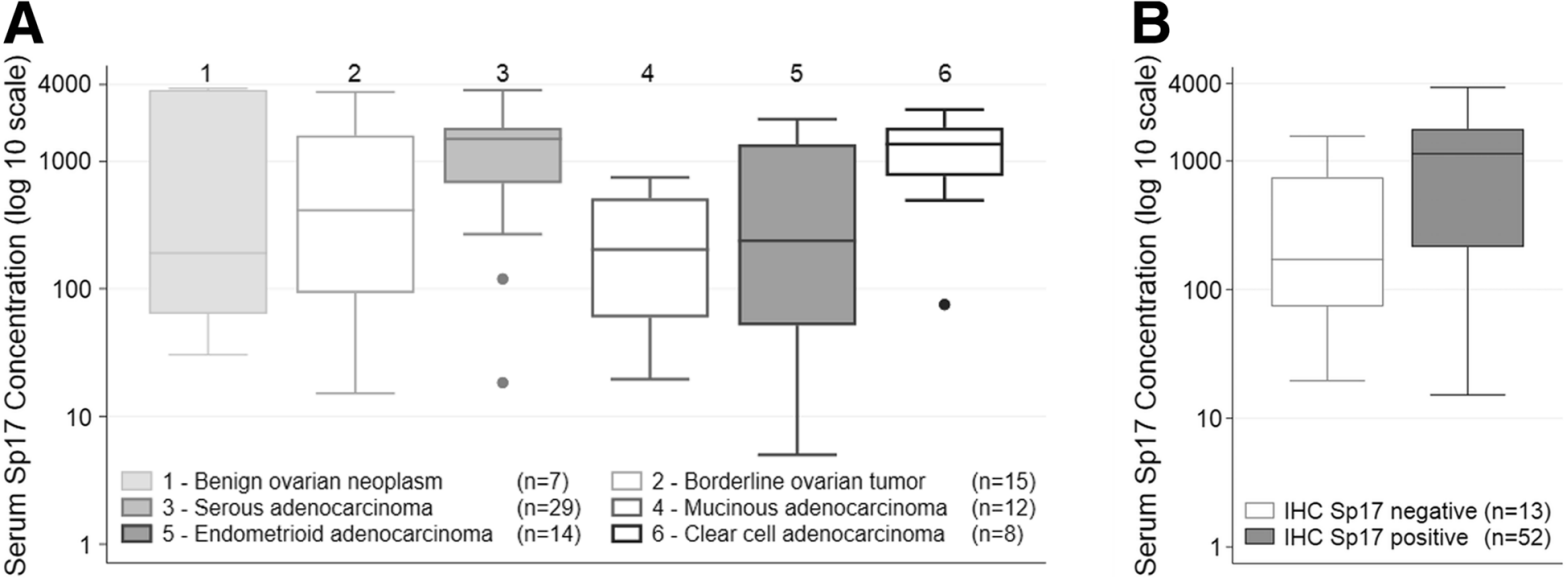
Serum Sp17 concentration compared to histology and tissue expression. **a** Distribution of serum Sp17 concentration in benign ovarian neoplasms, borderline ovarian tumors (BOTs), and epithelial ovarian carcinomas (EOCs). The serum concentration of Sp17 is plotted in standard box and whisker plots on a log 10 scale by histology. No significant difference was found between the serum Sp17 concentration when comparing benign ovarian neoplasms, BOTs, and EOCs. ANOVA test showed that there was a significant difference (p < 0.001) in serum Sp17 concentration among the four main histologic subtypes of EOC: serous, mucinous, endometrioid, and clear cell. Box (interquartile range) encompasses 25th–75th percentile, solid black line indicates median and the whiskers indicate the lowers and highest data values that are still within the 25th and 75th percentile value plus 1.5 times the interquartile range, respectively. The outliers are represented as separate data points. Numbers in parentheses indicate number of cases included in the analysis. **b** Distribution of serum Sp17 concentration by tissue expression of Sp17. There were 65 patients with both serum and ovarian tissue available, which included benign, borderline, and malignant ovarian neoplasms. The ovarian tissue underwent immunohistochemical (IHC) staining for Sp17 and any expression was considered a positive result. The matching sera for these patients were analyzed for Sp17 concentration using ELISA and the concentration converted to a log 10 scale and represented here in box and whisker plots. There was a significant difference in serum Sp17 levels between IHC positive neoplasms compared to IHC negative neoplasms (*p* = 0.027), with IHC positive neoplasms showing higher SP17 levels. Box (interquartile range) encompasses 25th–75th percentile, solid black line indicates median and the whiskers indicate the range. Numbers in parentheses indicate number of cases included in the analysis

To determine whether Sp17 protein could be detected in the serum of patients with EOC, we analyzed Sp17 concentrations in patients with adnexal masses clinically suspicious for malignancy who underwent surgery at LAC+USC Medical Center. There were 104 patients with serum tested for Sp17 concentration using ELISA. The median Sp17 concentration across all specimens was 696 pg/mL (range: 5–3715 pg/mL). These data are summarized in Table 4 according to histology. No significant difference was found between the serum Sp17 concentration when comparing benign ovarian neoplasms, BOTs, and EOCs. There was a significant difference (*p* < 0.001) in serum Sp17 concentration among histologic subtypes of EOC (Fig. 3a). Serum Sp17 concentration was significantly higher in serous carcinomas than in mucinous (ratio 6.2, 95% CI 1.8–20.8) or endometrioid (ratio 4.5, 95% CI 1.4–14.2) carcinomas, and clear cell carcinomas showed significantly higher levels than mucinous carcinomas (ratio 5.9, 95% CI 1.2–29.5). No significant difference was found in the remaining histologic comparisons. Thus, serum Sp17 levels appear to be highest among women with serous or clear cell EOC. Of the 104 patients with serum Sp17 measurements, 103 had clinically available CA125 measurements and 40 had HE4 measurements. No significant correlation was found between Sp17 serum concentration and CA125 or HE4 serum concentrations (data not shown). There was also no significant correlation when this cohort was limited to only those with epithelial ovarian carcinoma.

**Table 4:** Median Sp17 serum concentration by type of ovarian neoplasm

Disease Category	N	Sp17 serum concentration (pg/mL), Median (range)
Benign ovarian neoplasms	7	191 (31, 3715)
Borderline ovarian tumors	15	413 (15, 3475)
Epithelial ovarian carcinomas	75	922 (5, 3597)
Serous	29	1499 (18, 3597)
Mucinous	12	206 (20, 747)
Endometrioid	14	250 (5, 2143)
Clear cell	8	1368 (76, 2547)
Mixed/Other	12	1015 (30, 2234)
Sex cord stromal tumors	5	41 (20, 591)
Germ cell tumors	2	- (95, 702)
*All ovarian neoplasms*	*104*	*696 (5, 3715)*

### Correlation between Sp17 tissue expression and serum concentration

If serum levels of Sp17 are high due to overexpression of protein by ovarian tumor, we hypothesized that there would be a direct correlation between tissue and serum detection in the same patient. Sixty-five patients had both serum and ovarian tissue available for testing. Of these, 46 were from patients with EOC. Overall, 80% of these patients had Sp17 positive tissue specimens, including 87% of the EOCs. The median Sp17 serum concentration in this subset of patients was 945 pg/mL (range 15–3715). There was a significant difference in serum Sp17 concentrations between Sp17 positive compared to negative tissue specimens (*p* = 0.027) (Fig. 3b). For negative samples, the median (25th percentile, 75th percentile) was 171 (74, 746). For positive samples, the median (25th percentile, 75th percentile) was 1138 (218, 1771). Sp17 serum concentration at the time of surgery was not associated with FFS (See Additional file 1: Figure S1) or OS (See Additional file 1: Figure S2). Although not statistically significant, there was a trend for patients with the lowest levels of Sp17 to have the best FFS. Upon exploratory analysis using the 25th percentile as a cut-point compared to values above the 25th percentile, we found the differences to be not statistically significant (*p* = 0.23).

## Discussion

This study is the largest to date that examines Sp17 expression in ovarian tissues including benign, borderline, and malignant ovarian neoplasms with detailed analysis of histology, grade, stage, and prognostic significance. Sp17 EOC tissue expression by immunohistochemistry reported in the literature varies widely. Straughn et al.*......* demonstrated Sp17 positivity in 16 of 18 (89%) specimens, Gjerstorff et al in 2 of 10 (20%), and Vermeij et al in 173 of 270 (69%) [6, 10, 11]. No analyses were provided in these studies regarding histology, grade, or stage of the cancers with respect to Sp17 expression, likely due to the limited power with such small sample sizes. Li et al showed Sp17 expression in 30 of 70 (43%) EOC specimens with serous, mucinous, clear cell, and endometrioid carcinomas expressing 41, 35, 50, and 50% respectively [9]. Our study included 773 EOC specimens with 30% expressing Sp17. The differences in Sp17 expression seen across studies are likely multifactorial. We excluded patients who had been previously treated with chemotherapy, but the inclusion of these patients in other studies may account for differences in tumor gene expression profiles. For example, of the largest previously reported cohort of 270 patients, 85% had previously received chemotherapy, and the overall Sp17 tissue expression was higher at 69% [11]. We also had relatively low numbers of grade 1 and 2 cancers compared to the general population and these lower grade tumors were more likely to be Sp17 positive. Additionally, use of different Sp17 antibody clones for IHC staining between this and previous studies could affect staining patterns depending on the availability of antibody epitopes, although our antibody was validated using both positive and negative controls. We also tested multiple monoclonal and polyclonal primary antibodies, and the one used in our final protocol yielded the most specific staining. Additionally, 92% of tissue specimens in this study were represented on TMAs, and there is a possibility of false negatives compared to whole tissue specimens used in other studies as a result of sectioning. However, in the whole tissue specimens that we did test, Sp17 expression was evenly distributed throughout the tissue, even if less than 5% of cells stained positive. Despite these differences between patient populations and methodologies, our large sample size allowed us to identify specific tumor characteristics that correlated with higher expression of Sp17 with grade 1 cancers (40%) and serous neoplasms showing the highest expression. Among serous neoplasms, the highest expression was noted in serous cystadenomas (83%), BOTs (100%), and grade 1 carcinomas (51%) compared to higher grade serous carcinomas (32%). These specimens were both more likely to express Sp17 and more likely to have strong expression than the higher grade carcinomas.

There was significant overlap between Sp17 serum concentrations among patients with benign ovarian neoplasms, BOTs, and EOCs; however, among the EOCs, serous and clear cell carcinomas were found to have relatively higher serum concentrations than other histologies. This observation was supported by our examination of Sp17 mRNA levels reported from two large gene expression studies that were retrieved from the Oncomine database. There was also a correlation between patients with ovarian tissue Sp17 expression and higher Sp17 concentrations in serum, regardless of ovarian neoplasm type or histology. In this small sample size, we were not able to determine a cut-off value to distinguish between benign and malignant ovarian neoplasms or amongst the malignant histologies. These data suggest that Sp17 serum concentration might not be a useful general screening test in the absence of clinical symptoms for EOC given its low specificity. Future studies could evaluate the role of Sp17 alone or in combination with other known serum biomarkers for preoperative diagnosis. Our study also only evaluated Sp17 pre-treatment levels, but future studies evaluating the change in Sp17 concentration over time to monitor response to treatment or recurrence are needed to further elucidate the roles Sp17 may have as a biomarker for EOC.

Sp17 is normally expressed in ciliated epithelium throughout the body, including that of the fallopian tube epithelium, which is histologically similar to serous epithelial cells in ovarian neoplasms. Mounting evidence suggests that serous cystadenomas, serous borderline tumors, and low grade serous carcinomas are related and may arise from papillary tubal hyperplasia in the fallopian tube. High grade serous ovarian cancers are now considered a separate entity and may originate from serous tubal intraepithelial carcinomas [20–24]. These two classes of ovarian neoplasms have numerable differences in their genetic mutations and clinical behavior, however, they all share a common precursor in Sp17-positive serous epithelial cells. The decreasing Sp17 expression seen from benign to borderline to low grade serous carcinomas and the lowest expression seen in high grade serous carcinomas suggests a loss of expression of the cilia-related protein as cells become more poorly differentiated. An association between loss of cilia and decreased cilia-related gene expression has been found in other cancers including endometrial, renal cell, breast, melanoma, and pancreatic cancers [25–29]. Ciliary disassembly is induced by cellular stress, cell differentiation, and cell cycle progression. Thus, there is a known inverse relationship between cilia formation and cellular proliferation [26, 30]. A recent study of endometrioid endometrial cancers found that low expression of cilia-related genes correlated with higher grade tumors [38]. We similarly show in this study that the cilia-related protein, Sp17, shows decreased expression with increasing grade of EOC. It remains unclear whether cells that retain their Sp17 expression have a survival or metastatic advantage over Sp17-negative tumors or if this loss of Sp17 represents a normal physiologic change in carcinogenesis without other prognostic significance.

One small study showed that malignant cells in the ascites of eight EOC patients had 100% positivity for Sp17, as did ten metastatic tissues samples, although these data were not compared to non-metastatic tissue samples [9]. The authors also demonstrated an increased ability for tissue migration in Sp17 positive cells in an ovarian cancer cell model as well as an increased resistance to platinum chemotherapy agents among Sp17 positive cells. Another study of Sp17 expression in a clear cell ovarian cancer cell model found that Sp17 expression increased the cell’s resistance to paclitaxel chemotherapy [13]. Collectively, these prior studies propose a potential role of Sp17 in enabling metastases and/or resisting chemotherapy, suggesting a poorer prognosis for patients with Sp17-positive tumors. Our study did not find a correlation between Sp17 tissue expression and cancer stage, which would have been expected based on these prior studies. There was also no correlation between Sp17 positivity and FFS or OS on univariate or multivariable analyses. Furthermore, we found that Sp17 had higher expression in benign and borderline serous neoplasms compared to cancers, with further decreasing expression as the grade of the cancer increased. While serous BOTs and low grade carcinomas have an overall better prognosis than high grade carcinomas, they are known to be inherently chemoresistant neoplasms that are challenging to treat if complete surgical excision cannot be achieved [21–23, 31]. The relationship between Sp17 expression and lower grade that we have demonstrated may explain why previous studies have shown a correlation between higher Sp17 expression and chemoresistance and why we do not observe a poorer prognosis among Sp17 positive patients. Given the disappointing responses to chemotherapy in these patients, if an effective targeted therapy is encountered against Sp17, identifying patients who express this protein may purport a survival benefit. Chiriva-Internati et al. demonstrated that Sp17 could be used as a tumor vaccine against Sp17-positive ovarian cancer cells [12], and his group later described a significant survival benefit in a murine ovarian cancer model after administration of either a prophylactic or therapeutic anti-Sp17 vaccine [32]. These initial results are promising and further studies on Sp17 as a therapeutic target should be pursued.

A major strength of this study is the large number of specimens analyzed compared to prior studies. This is also the first study to measure Sp17 concentration in serum and the first to directly correlate serum and tissue levels in ovarian cancer patients. To our knowledge, no other studies of Sp17 expression in ovarian cancer have included other clinical data such as stage, grade, and survival outcomes. However, this study was limited in that not all specimens had complete clinical data and the number of patients with both serum and tissue for analysis was small. By including both benign and malignant ovarian neoplasms of all histologies, grades, and stages, we were able to analyze and compare Sp17 expression across all subtypes of adnexal masses and establish that Sp17 expression may have diagnostic implications in a subset of ovarian neoplasms. However, the variety of specimens included limited further subgroup analysis except among the serous adenocarcinomas, as this is the most common subtype of EOC, and made up a substantial portion of our specimens. Finally, our results were reported using the Silverberg three-tiered grading system as this was the system in use at the time most of the specimens were collected. The two-tiered grading system is now most commonly used, but it was not possible to reassign a grade on the limited TMA specimens. Future studies are warranted on specimens graded using the two-tiered system to validate these findings.

## Conclusions

In summary, Sp17 is highly expressed in benign and borderline serous ovarian neoplasms and grade 1 serous adenocarcinomas as well as other grade 1 epithelial ovarian carcinomas. The function and prognostic significance of Sp17 expression remains unclear, but this study identifies subsets of patients with BOTs and EOCs that are more likely to express the protein which may ultimately be found to have prognostic implications. Sp17 was not shown in this study to be a clinically useful screening biomarker for ovarian cancer, but we did find that Sp17 serum levels correlate with tissue expression. Additional studies are needed to analyze Sp17 alone and in ovarian cancer tumor marker panels to determine its utility in diagnosis or monitoring for recurrence or progression.

## Additional file

Additional file 1: **Table S1.** Progression free survival among patients with epithelial ovarian cancer. Table S2 Overall survival among patients with epithelial ovarian cancer. Figure S1 Figure of failure free survival among patients with epithelial ovarian cancer by Sp17 serum concentration. Figure S2 Figure of overall survival among patients with epithelial ovarian cancer by Sp17 serum concentration. (PDF 359 kb)

## Abbreviations

ANOVA: Analysis of Variance
BOT: Borderline ovarian tumor
CA-125: Cancer antigen 125
CI: Confidence interval
CTA: Cancer-testis antigen
ELISA: Enzyme-linked immunosorbent assay
EOC: Epithelial ovarian cancer
FFS: Failure free survival
GCT: Germ cell tumor
HE4: Human epididymis protein 4
HR: Hazard ratio
IHC: Immunohistochemical staining
mRNA: Messenger ribonucleic acid
OR: Odds ratio
OS: Overall survival
SCST: Sex cord stromal tumor
Sp17: Sperm protein 17
TMA: Tissue microarray
