# Supplementary material for: Validity and prognostic significance of sperm protein 17 as a tumor biomarker for epithelial ovarian cancer: a retrospective study
Source: BMC Cancer. 2018 Oct 11;18:970. doi: 10.1186/s12885-018-4880-x (PMC6182788; doi:10.1186/s12885-018-4880-x)
Supplement: Supplementary file 1 — Table S1. Progression free survival among patients with epithelial ovarian cancer. Table S2 Overall survival among patients with epithelial ovarian cancer. Figure S1 Figure of failure free survival among patients with epithelial ovarian cancer by Sp17 serum concentration. Figure S2 Figure of overall survival among patients with epithelial ovarian cancer by Sp17 serum concentration. (PDF 359 kb) [file 12885_2018_4880_MOESM1_ESM.pdf]

Brunette et. al., ADDITIONAL FILE 1

Table S1: Progression free survival among patients with epithelial ovarian cancer.

Variable	N=326	Univariate		Multivariable [3]	
		HR[5] (95%CI)	p	HR[5] (95%CI)	P
**Age at Diagnosis (years)**			0.008 [2]		0.73 [2]
0-39	19	1.0		1.0	
40-49	56	4.2 (1.6, 11)		2.0 (0.72, 5.6)	
50-59	96	2.4 (0.96, 6.1)		1.5 (0.54, 4.0)	
≥ 60	154	3.8 (1.5, 9.4)		1.8 (0.67, 4.8)	
Unknown[4]	1				
**Grade**			<0.001 [2]		0.13 [2]
1	36	1.0		1.0	
2	60	3.1 (1.3, 7.0)		1.8 (0.75, 4.5)	
3	230	4.2 (1.9, 9.0)		2.1 (0.88, 4.9)	
**Stage**			<0.001 [2]		<0.001 [2]
I	72	1.0		1.0	
II	27	1.1 (0.42, 2.9)		1.5 (0.53, 4.1)	
III	195	5.9 (3.2, 11)		6.0 (3.0, 12)	
IV	32	9.4 (4.8, 18)		9.9 (4.5, 22)	
**Histology**			<0.001 [1]		0.026 [1]
Serous	207	1.0		1.0	
Mucinous	15	0.56 (0.21, 1.5)		2.8 (0.88, 9.1)	
Endometrioid	35	0.30 (0.16, 0.57)		0.45 (0.23, 0.88)	
Clear cell	37	0.55 (0.32, 0.92)		1.1 (0.62, 1.9)	
Mixed/Other	32	0.78 (0.46, 1.3)		0.78 (0.46, 1.3)	
**Sp17 expression by IHC**			0.69 [1]		0.33 [1]
Negative	187	1.0		1.0	
Positive	139	0.93 (0.67, 1.3)		0.82 (0.55, 1.2)	

Abbreviations: HR – Hazard Ratio; CI – Confidence Interval; IHC - Immunohistochemistry

[1] Overall p value

[2] p value from trend test.

[3] Multivariable model includes all variables listed in the table.

[4] Patients with unknown values were included in the analysis using an unknown category.

[5] Hazard ratio calculated based on the regression parameter estimates in the survival model.

Table S2: Overall survival among patients with epithelial ovarian cancer.

Variables	N=336	Univariate		Multivariable [3]	
		HR[5] (95%CI)	p	HR[5] (95%CI)	P
**Age at Diagnosis (years)**			0.095 [2]		0.97 [2]
0-39	19	1.0		1.0	
40-49	57	3.8 (1.1, 13)		2.0 (0.55, 7.5)	
50-59	100	2.3 (0.72, 7.6)		1.4 (0.41, 5.0)	
≥ 60	159	3.3 (1.04, 11)		1.9 (0.55, 6.8)	
Unknown [4]	1				
**Grade**			0.006 [2]		0.080 [2]
1	36	1.0		1.0	
2	62	1.9 (0.63, 5.8)		1.4 (0.44, 4.5)	
3	238	2.9 (1.1, 8.0)		2.1 (0.68, 6.3)	
**Stage**			<0.001 [2]		0.010 [2]
I	73	1.0		1.0	
II	28	1.6 (0.43, 5.6)		2.0 (0.53, 7.8)	
III	201	4.1 (1.6, 11)		3.9 (1.4, 11)	
IV	34	5.1 (1.8, 14)		4.4 (1.5, 13)	
**Histology**			0.20 [1]		0.39 [1]
Serous	213	1.0		1.0	
Mucinous	16	1.3 (0.42, 4.3)		4.3 (1.2, 15)	
Endometrioid	35	0.54 (0.26, 1.1)		0.97 (0.45, 2.1)	
Clear cell	40	0.64 (0.34, 1.2)		1.0 (0.54, 2.0)	
Mixed/Other	32	1.2 (0.67, 2.1)		1.2 (0.67, 2.2)	
**Sp17 expression by IHC**			0.57 [1]		0.94 [1]
Negative	195	1.0		1.0	
Positive	141	0.88 (0.57, 1.4)		0.98 (0.62, 1.6)	

Abbreviations: HR – Hazard Ratio; CI – Confidence Interval; IHC - Immunohistochemistry

[1] Overall p value

[2] p value from trend test.

[3] The multivariable model included all the variables listed in the table.

[4] Patients with unknown values were included in the analysis using an unknown category.

[5] Hazard ratio calculated based on the regression parameter estimates in the survival model.

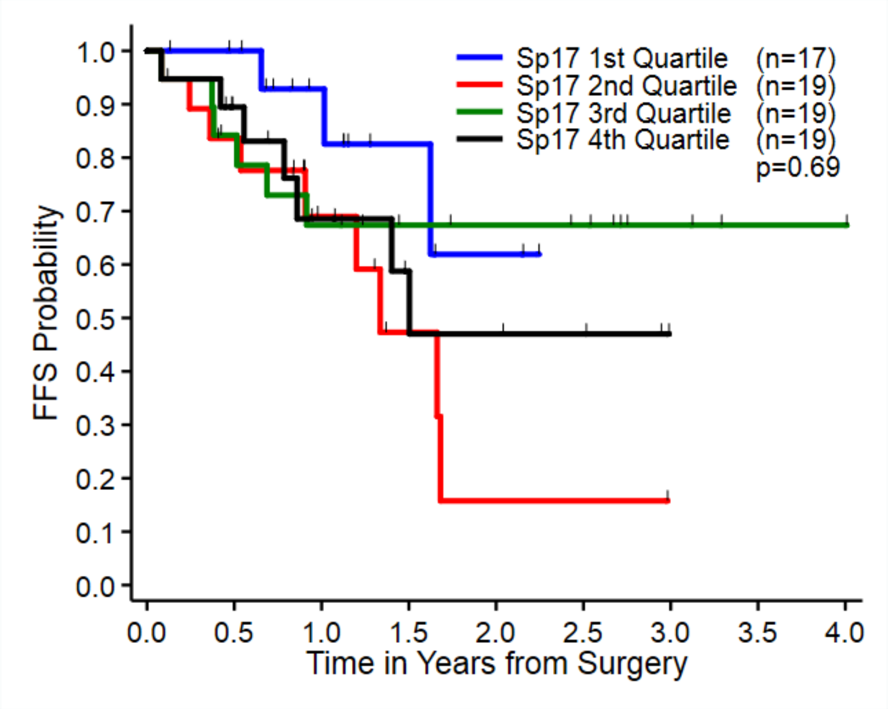

Figure S1. Failure free survival (FFS) of patients with epithelial ovarian cancer (EOC) by Sp17 serum concentration. All 75 patients with EOC with serum available underwent quantification of Sp17 concentration using ELISA. Kaplan-Meier curves were constructed to show FFS measured from time of surgery to time of recurrence or death, if death occurred prior to recurrence as defined in materials and methods. Recurrence status was unknown for one patient and she was excluded. Sp17 concentration was broken into quartiles. There was no association between serum concentration and FFS ($p = 0.69$).

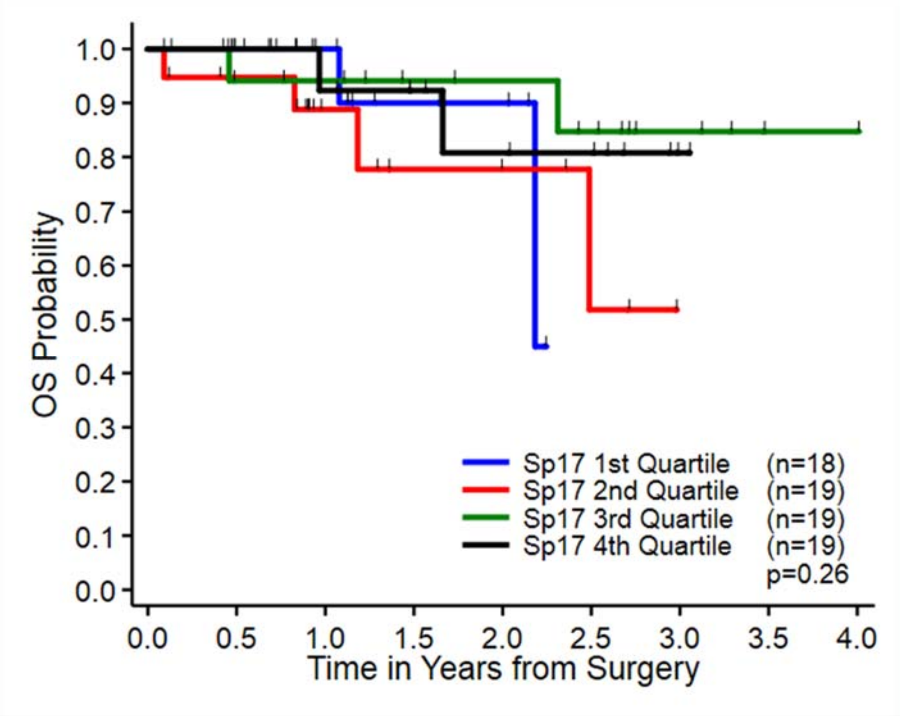

Figure S2: Overall survival (OS) of patients with epithelial ovarian cancer (EOC) by Sp17 serum concentration. All 75 patients with epithelial ovarian cancer (EOC) with serum available underwent quantification of Sp17 concentration using ELISA. Kaplan-Meier curves were constructed to show OS measured from time of surgery to time of death by any cause. Sp17 concentration was broken into quartiles. There was no association between serum concentration and OS ($p = 0.26$).
